# Evaluation of Cyclosporine Pharmacokinetic, Monitoring, and Dosing Parameters for GVHD Prophylaxis in Hematopoietic Stem Cell Transplant (HSCT) Recipients

**DOI:** 10.22037/ijpr.2020.112111.13539

**Published:** 2019

**Authors:** Ali Tafazoli, Simin Dadashzadeh, Mahshid Mehdizadeh, Sayeh Parkhideh, Maria Tavakoli-Ardakani

**Affiliations:** a *Student’ Research Committee, School of Pharmacy, Shahid Beheshti University of Medical Sciences, Tehran, Iran.*; b *Department of Pharmaceutics, School of Pharmacy, Shahid Beheshti University of Medical Sciences, Tehran, Iran.*; c *Pharmaceutical Sciences Research Center, Shahid Beheshti University of Medical Sciences, Tehran, Iran.*; d *Department of Bone Marrow Transplant, Taleghani Hospital, Shahid Beheshti University of Medical Sciences, Tehran, Iran.*; e *Department of Clinical Pharmacy, School of Pharmacy, Shahid Beheshti University of Medical Sciences, Tehran, Iran.*

**Keywords:** Cyclosporine, Pharmacokinetics, Hematopoietic stem cell transplantation, Graft versus host disease, Oral, Intravenous

## Abstract

Allogeneic hematopoietic stem cell transplantation (AHSCT) is a major method of treatment for different hematologic and congenital disease. Graft versus host disease (GvHD) is a life-threatening adverse effect of AHSCT. Cyclosporine is the most important and common agent for GvHD prophylaxis. Because of variable and unpredictable pharmacokinetics of cyclosporine that produces different responses in each patients group and clinical setting, there are still lots of uncertainties about its optimal method of administration and monitoring of this drug. Frequent blood samples in eight different times were taken for cyclosporine quantification in twenty AHSCT recipients and pharmacokinetic parameters determined in both intravenous (IV) and oral administration and monitoring parameters assessed accordingly. Of pharmacokinetic parameters mean ± SD area under concentration – time curve (AUC), clearance, and half-life were estimated to be 5492 ± 1596 ng.h/mL, 19.44 ± 6.61 L/h, and 11.8 ± 5.4 h for IV and 7637.7 ± 2739.8 ng.h/mL, 19.42 ± 6.62 L/h, and 11.16 ± 5.9 h for oral administration, respectively. Appropriate oral to intravenous dosing ratio found to be about 1.6. Of monitoring parameters, C_0.5_ h and C_6_ showed the highest coefficient of determination for regression between single points and total area under curve. Evaluation of pharmacokinetic parameters derived from concentration versus time curve showed that the appropriate oral/IV is 1.6 for maintenance GvHD prophylaxis for outpatients could be helpful. Cyclosporine plasma concentration at 0.5 and 6 h after IV administration showed the highest correlation with AUC of this drug.

## Introduction

Cyclosporine (CsA) is the most widely utilized immunosuppressant in allogeneic hematopoietic stem cell transplantation (HSCT) for graft versus host disease (GVHD) prophylaxis. Despite widespread and long history of its application, there are lots of uncertainties about its optimal method of administration. CsA pharmacokinetics has a major role in this issue (1). CsA pharmacokinetics exhibited enormous alterations with marked inter and intra-patient variability for drug absorptions, distribution, clearance and elimination that makes generalization of data for a specific population to others or individuals, impossible (2, 3). Many efforts have been made to determine the pharmacokinetic parameters of CsA but almost always different values have been reported for them by each group of authors. Notably, results of solid organ transplantation studies, that are more abundantly available, are not superimposable to HSCT populations. Bone marrow transplant (BMT) recipients have unique specifications that demands distinct therapeutic strategies. For instance, a major difference of HSCT recipients is inflammatory and destructive damages to GI structure and function due to conditioning chemotherapy, GVHD, and high rates of GI viral infections (4, 5). All these factors made further investigations of CsA pharmacokinetic parameters specifically for HSCT populations more valuable.

Complexity of CsA pharmacokinetics also affects other aspects of its administration such as therapeutic drug monitoring and switching between dosage forms of IV to oral. The optimal therapeutic monitoring approach of CsA would be a method that closely predicts patient clinical outcome, and it needs to be reliable, applicable and compliable. Currently, many monitoring methods have been proposed in literature but unfortunately, there is no consensus for the best practice especially in HSCT setting. The area under blood concentration-time curve (AUC) could be the most important index in therapeutic drug monitoring of cyclosporine by showing the extent of exposure. Determination of cyclosporine AUC is not always applicable due to requirement for close and frequent blood samplings and consequently ethical, practical, and economical issues. Therefore, limited sampling strategies (LSS) that have been developed to predict the AUC_0–12h_ especially in solid organ transplantation, provide a great opportunity for optimal management of cyclosporine consumers. An LSS (or a sparse sampling strategy) is a technique aiming to estimate a pharmacokinetic parameter using a small number of samples. Modeling the relationship between the pharmacokinetic parameter (AUC in here) and the drug concentration at various times allows reduction in the number of samples required. The model can then be used to choose the best sampling times to determine the parameter accurately and precisely. The development LSS requires full pharmacokinetic profiles drawn with sufficient points to measure AUC accurately. The AUC is considered to be the dependent variable; the independent variables are the blood concentrations at each time point. Then an equation will be defined giving the AUC as a function of one or several concentrations.

Unfortunately, despite the development of several equations to estimate AUC by this approach, most of them don’t have the validity for potentially globalized use (4). Therefore, provision of accredited equations for estimation of AUC through controlled and goal directed studies for marrow transplant patients could be highly valuable for each center.

Another major concern especially in primary phases of HSCT is the optimal CsA dosing for GVHD prophylaxis which could be highly pharmacokinetic dependent. Many and also different recommendations have been proposed for this issue that could be a consequence of different administration approaches applied in different centers and studies. Among dosing complexities, one of the most important controversies is the standard dose ratio of intravenous to oral formulation switching during maintenance therapy. Recently, the twice daily administration through short infusions became more appealing in transplant centers at the start of GVHD prophylaxis (6). Patients with the lowest blood CsA concentration (<200 ng/mL) in the third weeks after transplantation have higher risk of incidence of GVHD (grade II-IV) in HSCT recipients (7). Currently, there are limited numbers of studies that investigate the optimal transition approach to oral dosage forms while this type of administration is implemented. In general, such trials had small study population. Therefore, evaluation and optimization of this challenge would be highly beneficial in management of increasing numbers of HSCT patients.

In this study, we tried to address all these controversies in a single population of HSCT patients to gain a more comprehensive concept. One of the most precise AUC sampling patterns was applied both at intravenous and oral methods of administrations for cyclosporine. In addition, we tried to enroll as much as possible, homogenous patients during our time-limited project in order to propose an acceptable intravenous to oral dose ratio through the calculation of cyclosporine bioavailability in patients of our center. 

## Experimental

Between April 2013 and July 2014, we enrolled allogeneic hematopoietic stem cell transplant recipients admitted in Taleghani, Bone Marrow Transplantation Center. All clinically stable transplant candidates with related or unrelated, match or non-match donor were evaluated consecutively for enrollment by inclusion and exclusion criteria. Inclusion criteria included age between 18 and 65, stable kidney function (baseline serum creatinine lower than 2), no liver dysfunction (lower than 2 times upper limits of normal for transaminases and bilirubin), feasibility of oral intake, taking standard disease specific conditioning regimen of our ward (busulfan/cyclophosphamide base), taking standard CsA based immunosuppression for GVHD prophylaxis. Exclusion criteria included uncontrolled infection or sepsis on admission, inability for oral intake, mental or psychiatrically uncooperative patients, pregnant or lactating patients, and HIV+ patients. The study was approved by Ethics Committee of Shahid Beheshti University of Medical Sciences. Written informed consent was obtained from all patients participating in the study.

*Cyclosporine administration*

All patients received cyclosporine from 2 days before transplantation for GVHD prophylaxis. The starting dose was 1.5 mg/kg/doses every 12 h by a 2 h infusion in normal saline solution. This dosage has been modified based on routine CsA level trough monitoring to maintain levels between 200 to 400 ng/mL (8-25). When patients have gained the ability to tolerate oral CsA, after resolution of chemo-related mucositis and start of adequate oral intake, the drug converted to oral dosage form with a 2.5 oral to intravenous dosing ratio. The median duration from transplantation to the switch day was about 13 days.

*Monitoring*

Cyclosporine trough levels were taken just before morning doses and analyzed twice weekly or more frequently in patient who shown symptoms of toxicity. During intravenous administration, when we were sure of reaching steady state CsA levels by our available trough levels and generally after passing more than 5 days of the last CsA dose modification, a whole 12 h AUC profile was taken through frequent central venous line blood sampling between two consecutive doses at 0, 0.5, 1, 1.5, 2, 2.5, 3, 3.5, 4, 6, 8, 10, and 12 h after the start of administration.

After transition to oral dosage form, again on steady state, another similar profile was taken just before patient discharge day.

*Sampling*

Blood samples were taken from central venous line multiple lumen catheters. One of the catheter lumens was devoted for blood sampling and the other one for drug administration. At first, a 5 mL mixture of heparin and normal saline solution was injected into the catheter. Thereafter, 10 mL of catheter content was withdrawn but kept aside in aseptic status. Thereafter, 2 mL of blood was withdrawn with another syringe and transported to the test tube. Then, the preserved 10 mL was infused back into the catheter and line clamped again. All blood samples were collected in EDTA anti-coagulated test tubes and stored in fridge (4 °C) then analyzed for CsA content quantification in less than 12 h with EMIT assay.

*Pharmacokinetic analysis*

According to CsA blood levels, the concentration – time curves were demonstrated. Area under curve was calculated with trapezoidal rule. In order to obtain dose corrected values, PO to IV dose ratio was used with Equations 1 and 2.

Equation 1.

$\mathrm{Corrected AUC}=\frac{\mathrm{AUC}(\mathrm{PO})}{\mathrm{PO to IV dose ratio}}$

Equation 2.

$\mathrm{Corrected Cmax}=\frac{\mathrm{Cmax}(\mathrm{PO})}{\mathrm{PO to IV dose ratio}}$

CsA oral bioavailability was calculated with the following formula which is frequently applied in literature (6, 7).

Equation 3.

$F=\frac{\mathrm{AUCpo}}{\mathrm{AUCiv}}\times \frac{\mathrm{DOSEiv}}{\mathrm{DOSEpo}}$

Elimination rate constant (K_el_) was derived from elimination phase of CsA concentration time curves and consequently clearance and terminal volume of distribution (V_d_) was calculated with the following formulas:

Equation 4.

Cl=F×Dose AUC

Equation 5.

$\mathrm{Vd}=\frac{\mathrm{Cl}}{\mathrm{Kel}}$

Also, CsA half-life was calculated as follows:

Equation 6.

$\mathrm{t1}/2=\frac{0.693}{\mathrm{Kel}}$

All calculations were carried out for both IV and oral CsA administration methods.

*Statistical analysis and limited sampling strategy*

The IBM SPSS^®^ statistics version 19 was applied to perform the statistical analysis. Also, we used Microsoft^®^ Excel to draw plots and calculate area under curve values.

Considering our limited number of patients, besides statistical evaluation of correlations between AUC or trough concentrations and events like adverse reactions, these markers categorized in quartiles in order to visually present the fact of probable associations between higher quartiles and more severe toxicities. For evaluation of statistical significance of correlations between categorical clinical variables with trough and AUC, Pearson’s chi-square was evaluated.

Because only 20 patients were evaluated in this study, Shapiro–Wilk test was used for normality of data distribution. For evaluation of correlations between two quantitative variables, Bivariate correlation was applied.

To develop the LSS, we checked all the possible correlations between monitoring parameters at different time points and AUC. All points were inserted individually and in combinations, one by one. For determination of the best concentration – time points that have the best correlations with AUC_0-12h_, linear regression between drug concentrations at each time point as independent variable and total AUC as dependent variable were assessed. Stepwise multiple linear regression also was applied for correlations between multiple points and total AUC. Afterwards, the reliability of the correlations was evaluated by production of Bland-Altman plot. Considering the limitations of SPSS for Bland-Altman plot production, especially for multiple variables, Bland-Altman plotting was undertaken manually only for chosen points with acceptable regression correlations and potential clinical applicability. All statistical analysis of differences between the samples was performed using sample *t*-test. *P* < 0.05 was considered statistically significant. Data were reported as mean ± SD.

## Results

Among the patients, 11 were female and 9 were male. The median age for our study population was 27 years (min: 21 and max: 50 years). Mean weight was 67.7 ± 14.1 kg and mean height was 1.66 ± 0.11 m (mean ± SD). The underlying diseases were acute myeloid leukemia (n = 12), acute lymphoblastic leukemia (n = 6), non-Hodgkin lymphoma (n = 1), and aplastic anemia (n = 1). All patients received peripheral blood hematopoietic stem cells from match related donors. The median durations from transplantation day to profile sampling for IV and PO AUC estimations were about 7 days (range: 5–10 days) and 15 days (range: 12–21 days), respectively. Mean 12 h AUC for twice daily intravenous and oral administrations were 5492 ± 1596 and 7637.7 ± 2739.8 ng.h/mL (mean ± SD), respectively; In order to compare IV and PO parameters, dose corrected values were also calculated for oral parameters. Dose corrected AUC for PO profile was 3769.5 ± 1621.9 ng.h/mL (mean ± SD). C_max_ for intravenous profile was 1384 ± 412.8 ng/mL and for oral profile, it was 1369.3 ± 419 ng/mL (mean ± SD) (Dose corrected PO C_max_ = 680.9 ± 281.9 ng/mL). AUC_IV _was significantly higher than dose corrected AUC_PO_ (*P* = 0.001). Moreover, IV C_max_ was significantly higher than dose corrected PO C_max_ (*P* < 0.005). Mean IV dose at profile sampling time was 1.45 ± 0.1 mg/kg and mean PO dose was 3.15 ± 0.7 mg/kg (mean ± SD). When two unrealistic out layer values (F = 2.01, 1.68) were omitted, mean bioavailability was 0.61 ± 0.16 (min: 0.33, max: 0.96). Other pharmacokinetic parameters were also estimated and can be found in Table 1.

Full pharmacokinetic proﬁles of CsA were obtained for twenty patients both at IV and PO state. The linear regression equations and the concentration at each time point are listed in Tables 2 and 3.

The 10 models that utilized CsA concentrations at a single time point generally did not have a good ﬁt for IV administration (R^2 ^< 0.85). Highest R^2^ was detected for IV C_0.5_ (R^2^ = 0.77). Interestingly, for oral administration, only C_6_ showed acceptable R^2^ values (R^2^ = 0.86). Notably, the correlation between AUC_0–12h_ and the trough or peak concentrations were poor for both oral and IV administrations.

Stepwise multiple linear regression analysis of the correlation between the estimated cyclosporine (CsA) AUC_0–12h_ and all concentration–time points are listed in Table 4.

Also, the equations delineated from all of the concentration-time points together are shown below.

AUC_IV _= 5.044 × 10^-12 ^+ 25C_0 _+ 0.5C_0.5 _+ 0.5C_1 _+ 0.5C_1.5 _+ 0.5C_2 _+ 0.5C_2.5 _+ 0.5C_3 _+ 0.5C_3.5 _+ 1.25C_4 _+ 2C_6 _+ 2C_8 _+ 2C_10 _+ C_12_

R^2^ = 1

AUC_PO _= 1.989 × 10^-12 ^+ 0.250C_0 _+ 0.5C_0.5 _+ 0.5C_1 _+ 0.5C_1.5 _+ 0.5C_2 _+ 0.5C_2.5 _+ 0.5C_3 _+ 0.5C_3.5 _+ 1.25C_4 _+ 2C_6 _+ 2C_8 _+ 2C_10 _+ C_12_

R^2^ = 1

Among models that used CsA concentrations at 2 time points from C_0_ to C_4_, best model was AUC = 310.422 + 2.296C_2 _+ 5.226C_4_, (R^2 ^= 0.904) for IV and AUC = 664.116 + 3.599C_3.5 _+ 8.035C_8_, (R^2 ^= 0.959) for PO profile.

One by one inclusion of concentration – time points from C_0_ to C_12 _in regression equations revealed that the first model that reaches an accepTable R squared (R^2 ^> 0.85) is AUC = 515.297 + 4.390C_0 _+ 0.176C_0.5 _+ 3.245C_1 _- 3.876C_1.5 _+ 2.014C_2 _+ 3.682C_2.5_ for IV and AUC = 514.249 + 4.7C_0 _+ 1.667C_0.5 _+ 0.792C_1 _- 1.111C_1.5 _- 0.264C_2 _+ 4.554C_2.5_ for PO profile. In these results, 2.5 h post dosing was a common point for both administration routes.

By limiting optimal limited sampling strategy equations to those using a maximum of two concentration–time points obtained within 4 h after start of dosing, and with a coeffcient of determination >0.85, AUC = 310.422 + 2.296C_2_ + 5.226C_4_, R^2 ^= 0.904 for IV and AUC = 790.985 + 7.086C_0.5_ + 4.516C_4_, R^2^ = 0.926 were selected.

Beside concentration-time points, AUC fragment provides the chance for finding statistically significant correlations through regression without requirement for additional clinical or practical interventions (*e.g.* increased number of samplings) only by utilizing mathematical calculations on the same data set.

Evaluation of regression between total AUC and cumulative AUC fragments from C_0_-C_0.5_ to C_10_-C_12_ showed that the first cumulative fragment with R^2 ^> 0.85 is AUC_0-6_ for IV and AUC_0-4 _for PO profile (AUC_0-12h _= 243.862 + 1.377AUC_0-6h_, R^2^ = 0.922, AUC_0-12h _= -916.536 + 2.352AUC_0-4h_, R^2^ = 0.889, respectively).

Also among separate AUC fragments, AUC_6-8h_ for both IV and PO profile demonstrated the highest coefficient of determinations (AUC_0-12h _= 2200.352 + 5.268AUC_6-8h_, R^2^ = 0.695, AUC_0-12h _= 2012.650 + 5.396AUC_6-8h_, R^2^ = 0.896, respectively).

Considering the C_6_ point in oral adminis-tration as the only potential practically applicable monitoring parameter in our findings with acceptable statistical value (R^2^ = 0.86), we checked the reliability for the related equation. This parameter showed a non-significant difference between means of two measures (C_6_ AUC estimation and true AUC) by one sample *t*-test. The Bland-Altman plot showed only one out layer point. Also checking the linear regression for differences and mean values showed absence of significant proportional bias for C_6_ AUC estimation (*p*-value = 0.403).

From clinical observations, engraftment was achieved in all cases. Acute graft versus host disease was observed in two, one, three, and two patients in Glucksberg grade 1, 2, 3, and 4, respectively. Severity of acute GVHD was not statistically correlated to AUC or mean trough values. All cases were successfully treated with steroid therapy.

Possible CsA related toxicities were also evaluated and categorized according to “National Cancer Institution (NCI) Toxicity Criteria” for nephrotoxicity, neurotoxicity, hypertension, hepatotoxicity, and hyperglycemia as stated in Table 5.

There were no statistical correlations between occurrence or severity of these toxicities and CsA exposure markers, namely, C_trough_ and AUC.

## Discussion

Despite routine CsA utilization in transplant setting, the most appropriate method of monitoring and immunosuppressive strategy of dosing is still debated. One of the most important controversies of cyclosporine administration is the standard dose ratio of intravenous to oral formulation switching during maintenance therapy. As noted by EBMT–ELN guideline, the conversion factor varied majorly between 1 and 3 in literature (8). In this study, comparison between intravenous AUC or C_max_ with dose corrected oral values showed higher levels for IV method and this fact proposes that if we used 1 to 1 ratios, patients would suffer from considerable under-exposure during the initial phase of oral CsA administration. Such ratios are commonly developed by calculating bioavailability for CsA oral formulations in several studies (6, 9 and 10). It could be stated that utilizing bioavailability as guidance for this issue significantly requires individualization for specific formulations, brands and subject populations but the final guiding standard should always be monitoring of exposure-markers.

Parquet *et al.* were one of the first groups that tried to predict the best dosage of Neoral^®^ when patients are switched from IV to oral administration in bone marrow transplantation setting. Therapeutic goal was maintaining the blood trough CsA level within 150–250 ng/mL. Results demonstrated that the conversion in a dose ratio of 1 to 1 leads to under-exposure for some patients and the conversion in a dose ratio of 1 to 2 allows obtaining optimal therapeutic exposure (9). Dotti *et al.* also evaluated the pharmacokinetic profile of Neoral^®^ in 18 allo-BMT patients early after transplantation. Prophylaxis regimen for acute GVHD consisted of intravenous CsA 1 mg/kg/day over 24 h. Therapeutic range of 200-400 ng/mL was chosen on serum with a polyclonal-radioimmunoassay. They suggested 7.5 mg/kg/Day of Neoral^®^ as an appropriate starting oral dose of CsA, provided adequate CsA trough levels are maintained without significant acute renal toxicity after switching from intravenous form and this was in line with results of Parquet *et al.* in the setting of allo-BMT (11). In more recent trials, separation of study population into different dosing groups for transition, changed to evaluation of one group with estimation of the optimal ratio through IV and PO pharmacokinetic data.

In a study carried out by Kimura *et al.*, the authors focused on the transition period more closely. Patients switched to oral administration at a dose ratio of 1:2 as they acquired the ability to tolerate oral intake and intravenous infusion was stopped just before the ﬁrst oral administration. Bioavailability of Neoral^®^ was estimated by dividing (AUC _PO_/DOSE _PO_) by (AUC _IV_/DOSE _IV_) and the median value was 0.685 (10). Choi *et al.* also studied the transition period between continuous IV infusion and oral CsA treatment while applying a 3 to 1 ratio and evaluated the pharmacokinetic properties of CsA in 33 pediatric HSCT recipients with a median age of 7.1 years. The mean bioavailability of CsA in this pediatric population was 43.1 ± 14.4%, when compared to reported adult HSCT patients which was about 34%. An important finding of this study was shorter half-life and the higher clearance in pediatric population, which proposes a possible privilege of dosing every 8 h versus twice daily dosing in children as implemented in some centers (12, 13).

Inoue *et al.* stated in 2012 that the appropriate dose–rate conversion and target blood concentration for CsA has not yet been established in allo-HSCT. They noted the common phase for oral tolerability at about 2 to 4 weeks and a 1:2-3 dose conversion ratios according to literature. As an advantage for this study, they investigated the serial changes in the CsA blood concentration during the switch from 1.5 mg/kg twice daily “3 h short infusion”- which recently became more prevalent in use when compared to oral administration and estimated the bioavailability of Neoral^®^. Bioavailability of Neoral^®^ was 0.58 ± 0.15 (mean ± SD, range 0.41–0.94) so they concluded that the conversion ratio of 1:2 is appropriate (6). This ratio was also adopted by Eljebari *et al.* for oral CsA (Equoral^® ^or Neoral^®^) at twice IV dose in allogeneic hematopoietic stem cell transplantation recipients (4).

The mean value calculated for bioavailability in our study (F = 0.61) was in line with the above mentioned studies. Consequently, it could be stated that arbitrarily, an IV to PO ratio of 1 to 2 would be acceptable for this population. Indeed an index of 1.6 would suggest a more exact dosing ratio. Therefore, the empiric ×2.5 ratio which we applied seems to be excessive and could possibly result in toxicities, although there were no statistical correlations between severity of toxicities and extent of CsA exposure. In addition, it should be considered that according to high variations in the results among patients, these estimated ratios should never be substituted for close blood level monitoring.

As an advantage for our study, the minimum recommended time by manufacturer (2 h) for intravenous CsA infusions was implemented which could be important in time saving in this population that requires frequent daily administrations. Considering the fact that different modes of CsA infusion in each study complicate the determination of optimal approach (6), evaluation of pharmacokinetic parameters and dosing or monitoring markers for this specific and desirable method of administration would be highly valuable.

The optimal therapeutic monitoring approach of CsA would be a method that closely predicts patient clinical outcome, reliable, applicable and compliable. Currently, numerous monitoring methods have been proposed in literature but unfortunately, there is no consensus for the best practice especially in HSCT field. Lack of agreement among authors could be as a result of heterogeneity of related trials regarding applied analytical method (14, 15), type of biologic sample (15), time and pattern of sampling (4, 5 and 16-19), post-transplantation time (3), proximity of drug consumption to meals (1, 20), paucity of data in HSCT (2, 20-22), lack of validation for proposed approaches (14, 17), study defects or lack of power for such conclusions (20, 23), varied composition of study populations (20), and different therapeutic target ranges (11, 18, 20 and 23-27). Generally, it could be assumed that an optimal monitoring program will include fixed times for both dosing and sampling times and consistent status even regarding meal intake time to provide reproducible and evaluable results (1). Trough blood monitoring is routinely utilized for CsA therapeutic drug monitoring, but this approach is not optimal due to the fact that the area under the AUC showed better correlations with patient clinical outcomes. Common methods of measuring AUC require frequent blood sampling, which is not easily applicable in clinical practice. It has been demonstrated that AUC monitoring can be simplified by utilizing limited sampling strategies that allows AUC estimations with small number of blood samples obtained at specific times during a dosing interval (17). As mentioned before, the optimal LSS could be yielded after validation of the equations in a recruited validation group. There were barriers for doing so in this study. Our method of blood sampling in this study was considerably invasive and frequent. Ethical considerations in our center prevented us to implement such procedure on more than 20 patients. We didn’t want to decrease the value of the results by breaking the study population in two 10-member groups. Also there were time and budget limitations. Therefore, we forced to give up the external validation and provided the correlation results for better demonstration of our findings in a simple way. This defect could be stratified with a straightforward approach in future studies.

A major ﬁnding of our study is that a single concentration – time point measured at C_6 _in oral administration would presumably be an optimal surrogate indicator of AUC_0–12h_ in allogeneic hematopoietic stem cell transplant recipients with acceptable reliability. Schrauder *et al.* also found C_6_ with highest coefficient of determination (which was not acceptably adequate); but for CsA, it was 2 h infusion (R^2^ = 0.77) in 27 allo-HSCT pediatric patients. Unfortunately, we could not find similar results for single points in intravenous administration like this and many other studies (18) and the highest R^2^ belonged to the model including C_0.5_ (R^2 ^= 0.77). From multiple point models, our suggested equations with acceptable R^2^ and applicable sampling times included C_2_ and C_4_ for IV and C_0.5_ and C_4_ for PO profile. These models could be unique for this study design and this specific population.

Currently, it can be stated that more than hundreds of equations have been proposed for limited sampling strategy from the first attempts (4, 14 and 28). It is a scarce observation that different authors use or propose similar sampling times (17). As an exception, Dotti *et al.* applied a 3 point sampling strategy proposed by a kidney transplant study for allo-BMT patients and interestingly found an accurate prediction for AUC (11, 29). Also, Mahalati *et al.* determined AUC_0-4h_ utilizing a regression formula from their retrospective study database (26, 30). It has been stated that application of these limited sampling strategies should be dedicated to the population in which they were developed and not even with another analytical method (14, 17). Importantly, it should be stated that validating the equations on a second data set different from the training set as our population is obligatory for attaining reliable equations in limited sampling strategy (17). Due to limited number of patients in our study, inclusion of a testing set was practically impossible and this is a considerable limitation.

Clinical outcomes of patients were also observed and evaluated in this research. There was no statistically significant correlation between CsA exposure and severity of toxicities or GVHD. Variable and weak association of trough levels with clinical outcomes especially GVHD or its severity (3, 23 and 24) and lack of potential for prediction for adverse clinical outcomes (31) has been reported frequently. Also for AUC, the results were not always conclusive (23, 24). Contrary to findings that propose a concentration or pharmacokinetic dependent nature for CsA complications, some other observations also suggest that renal dysfunction could occur even with therapeutic blood concentrations of CsA and non-related to CsA use (32-34). CsA neurotoxicity could also be seen at both therapeutic and excessive CSA levels (35). Similar findings are available for CsA associated hypertension (36) and hyperglycemia (37). However, it should be noted that small sample size in this study would hinder a definite judgment. Due to the use of other nephrotoxic drugs (*e.g.* cyclophosphamide, amphotericin b, aminoglycosides, vancomycin), neurotoxic drugs (*e.g.* busulfan), hepatotoxic drugs (*e.g.* metothrexate, azoles), hyperglycemic agents (*e.g.* corticosteroids, parenteral nutrition), it can be difficult to assign these adverse effects to cyclosporine alone.

**Table 1:** Cyclosporine pharmacokinetic parameters in the study patients (mean ± SD, n = 20).

**Intravenous cyclosporine pharmacokinetic parameters**												
**AUC (ng.h/mL)**		**Cmax (ng/mL)**			**Clearance (L/h)**		**K**_el_ ** (h** ^-1^ **)**		**T**_1/2_ ** (h)**		**V**_d_ ** (L)**	
5492 ± 1596		1384 ± 412.8			19.44 ± 6.61		0.70 ± 0.02		11.8 ± 5.4		318.61 ± 151	
**Oral cyclosporine pharmacokinetic parameters**												
**AUC (ng.h/mL)**	**nAUC (ng.h/mL)**		**Cmax (ng/mL)**	**nCmax (ng/mL)**		**Tmax (h)**		**Clearance (L/h)**	**K**_el_ **(h** ^-1^ **)**	**T**_1/2_ **(h)**		**V**_d_ **(L)**
7637.7 ± 2739.8	3769.5 ± 1621.9		1369.3 ± 419	680.9 ± 281.9		2.27 ± 0.4		19.42 ± 6.62	0.78 ± 0.03	11.16 ± 5.9		313.78 ± 190.3

nAUC: dose corrected AUC; nCmax: dose corrected Cmax.

**Table 2:** Correlations and regression equations between AUC_0__−__12 h _and CsA IV concentrations

**Model**	**Concentration-time point**	**Equation**	**R** ^2^
1	C_0_	AUC = 4342.036 + 4.010C_0_	0.286
2	C_0.5_	AUC = 2203.718 + 5.008C_0.5_	0.770
3	C_1_	AUC = 2988.924 + 2.643C_1_	0.350
4	C_1.5_	AUC = 2152.93 + 2.797C_1.5_	0.439
5	C_2_	AUC = 1748.942 + 2.705C_2_	0.489
6	C_2.5_	AUC = 1988.206 + 5.443C_2.5_	0.494
7	C_3_	AUC = 2776.454 + 4.761C_3_	0.574
8	C_3.5_	AUC = 2719.926 + 5.942C_3.5_	0.555
9	C_4_	AUC = 3192.264 + 5.996C_4_	0.561
10	C_6_	AUC = 3013.268 + 7.533C_6_	0.532
11	C_8_	AUC = 3095.309 + 8.102C_8_	0.497
12	C_10_	AUC = 3398.904 + 8.401C_10_	0.479
13	C_12_	AUC = 4781.39 + 2.714C_12_	0.62

**Table 3:** Correlations and regression equations between AUC_0__−__12 h _and CsA PO concentrations

**Model**	**Concentration-time point**	**Equation**	**R** ^2^
1	C_0_	AUC = 4379.545 + 9.738C_0_	0.462
2	C_0.5_	AUC = 3736.985 + 10.209C_0.5_	0.554
3	C_1_	AUC = 4183.861 + 5.064C_1_	0.282
4	C_1.5_	AUC = 4565.899 + 3.211C_1.5_	0.198
5	C_2_	AUC = 2361.265 + 4.301C_2_	0.715
6	C_2.5_	AUC = 601.383 + 5.562C_2.5_	0.744
7	C_3_	AUC = 2472.542 + 4.644C_3_	0.580
8	C_3.5_	AUC = 2078.493 + 5.432C_3.5_	0.668
9	C_4_	AUC = 2350.588 + 5.768C_4_	0.692
10	C_6_	AUC = 2298.967 + 8.433C_6_	0.860
11	C_8_	AUC = 3006.418 + 11.311C_8_	0.726
12	C_10_	AUC = 3341.605 + 12.296C_10_	0.812
13	C_12_	AUC = 2712.570 + 16.404C_12_	0.617

**Table 4:** Stepwise multiple linear regression equations for AUC_0–12h_ and all monitoring points (N = 20).

**IV Profile**	**R** ^2^
AUC = 2203.713 + 5.008C_0.5_	0.770
AUC = 1518.112 + 3.786C_0.5_ + 2.610C_3_	0.896
AUC = 1374.446 + 3.324C_0.5_ + 2.140C_3_ + 2.172C_6_	0.922
AUC = 1263.021 + 2.613C_0.5_ + 1.909C_3_ + 2.466C_6_ + 2.462C_10_	0.946
AUC = 462.207 + 1.016C_0.5_ + 1.478C_3_ + 3.607C_6_ + 3.502C_10_ + 1.223C_1.5_	0.98
**PO Profile**	**R** ^2^
AUC = 2298.967 + 8.433C_6_	0.860
AUC = 1520.571 + 6.736C_6_ + 4.848C_0.5_	0.950
AUC = 897.340 + 4.188C_6_ + 5.327C_0.5_ + 2.240C_4_	0.985
AUC = 290.483 + 4.083C_6_ + 3.662C_0.5_ + 2.613C_4_ + 1.012C_1.5_	0.991
AUC = 90.783 + 2.762C_6_ + 1.966C_0.5_ + 3.083C_4_ + 1.253C_1.5_ + 2.498C_8_	0.996
AUC = -83.208 + 2.680C_6_ + 1.607C_0.5_ + 2.176C_4_ + 1.326C_1.5_ + 2.931C_8_ + 0.852C_3_	0.998
AUC = -228.624 + 2.244C_6_ + 1.286C_0.5_ + 1.845C_4_ + 1.189C_1.5_ + 3.248C_8_ + 1.033C_3_ + 0.512C_2.5_	0.999
AUC = -210.985 + 2.543C_6_ + 1.464C_0.5_ + 1.711C_4_ + 1.151C_1.5_ + 2.590C_8_ + 0.999C_3_ + 0.465C_2.5_ + 0.829C_12_	1
AUC = -128.524 + 2.618C_6_ + 1.326C_0.5_ + 1.617C_4_ + 1.126C_1.5_ + 2.084C_8_ + 0.872C_3_ + 0.572C_2.5_ + 0.814C_12_ + 0.720C_10_	1

**Table 5:** Frequencies of possible CsA toxicities in the study population based on NCI grading

**Toxicity Grade**	**0**	**1**	**2**	**3**	**4**
**Acute Kidney Injury**					
No of patients	16	1	2	1	0
**Neurotoxicity**					
No of patients	10	3	2	2	3
**Hypertension**					
No of patients	16	4	0	0	0
**Hepatotoxicity**					
No of patients	17	2	0	0	1
**Hyperglycemia**					
No of patients	1	2	8	8	1

## Conclusion

In conclusion, the appropriate dosing ratio of IV to PO cyclosporine for adult allogeneic hematopoietic stem cell transplantation patients, after accessibility of oral route post-transplant (after patent stabilization and resolution of conditioning mucositis), is 1.6 to obtain similar exposure by AUC values. It should be kept in mind that numerous accompanied situations by transplant process can alter this ratio very easily. Therefore, maybe using value “2” instead of “1.6” would be justifiable and also more memorable for clinicians. But for patients with drug elimination defects of any kind, values closer to “1.5” will be prudent to use. In addition, derived data from pharmacokinetic evaluations would help in the determination of limited sampling strategies to predict the total CsA AUC. Although several models for monitoring of AUC are proposed here, according the above-mentioned limits of our study, larger prospective studies are required to validate the monitoring approaches and assess correlations for clinical outcomes.
